# Ocular surface toll like receptors in ageing

**DOI:** 10.1186/s12886-022-02398-8

**Published:** 2022-04-22

**Authors:** Antonio Di Zazzo, Maria De Piano, Marco Coassin, Tommaso Mori, Bijorn Omar Balzamino, Alessandra Micera

**Affiliations:** 1 Ophthalmology Operative Complex Unit, University Campus Bio-Medico, Rome, Italy; 2 Research and Development Laboratory for Biochemical, Molecular and Cellular Applications in Ophthalmological Sciences, IRCCS–Fondazione Bietti, Rome, Italy

**Keywords:** TLRs, InflammAging, Para-inflammation, Ageing, Ocular Surface

## Abstract

**Background:**

To evaluate changes in Toll Like Receptors (TLRs) expression at the ocular surface of healthy volunteers within different age groups.

**Methods:**

Fifty-one healthy volunteers were enrolled in a pilot observational study. Clinical function tests (OSDI questionnaire, Schirmer test type I and Break Up time) were assessed in all subjects. Temporal Conjunctival imprints were performed for molecular and immunohistochemical analysis to measure TLRs expression (TLR2, 4, 3, 5, 7, 8, 9 and MyD88).

**Results:**

Immunofluorescence data showed an increased TLR2 and decreased TLR7 and TLR8 immunoreactivity in old conjunctival imprints. Up-regulation of TLR2 and down-regulation of TLR7, TLR8 and MyD88 transcripts expression corroborated the data. A direct correlation was showed between increasing ICAM-1 and increasing TLR2 changes with age. Within the age OSDI score increases, T-BUT values decrease, and goblet cells showed a decreasing trend.

**Conclusion:**

Changes in TLRs expression are associated with ageing, suggesting physiological role of TLRs in modulating ocular surface immunity. TLRs age related changes may participate to the changes of ocular surface homeostatic mechanisms which lead to inflammAging.

## Background

Ageing is a physiological process which lead to several structural and physiological changes. Particularly a dysregulation of the local innate and adaptive immune responses to environmental injuries has been proved [1]. A persistent subclinical low-grade inflammation has been demonstrated at ocular surface of elder self-considering ‘‘healthy’’ volunteers [2–4]. In those a dysregulation of para-inflammatory mechanisms, which keep tissue homeostasis against environmental injuries or insults, causes an ocular surface system failure, also defined InflammAging. Therefore ocular discomfort due to biomolecular impairments can seriously impact the quality of life of our older patients [2].

Epithelial cells also respond to stressors by releasing several inflammatory mediators which may influence the local immune state and overall tissue equilibrium [2]. In fact, corneal and conjunctival epithelial cells express numerous immune related recognition receptors, including Toll-Like Receptor (TLR) [5, 6]. TLRs actively participate in immunological tutoring and innate defense responses at ocular surface [5]. Particularly TLR1, TLR2, TLR4, TLR5, TLR6 and TLR11, which are expressed on cell surfaces, recognize mainly microbial membrane components such as lipids, lipoproteins and proteins, while TLR3, TLR7, TLR8 and TLR9, which are expressed exclusively in intracellular vesicles, such as the endoplasmic reticulum (ER), endosomes, lysosomes and endo-lysosomes, mainly link nucleic acids [7].

TLRs expression at epithelial and antigen presenting cells (APC) of the ocular surface seems to be relevant in driving innate/adaptive cross-talk of immune cells [5, 6]. In fact although TLRs are essential for protective immunity against infection, inappropriate TLRs responses contribute to acute and chronic inflammation, as well as to systemic autoimmune diseases. More importantly, evidence suggests their critical role in scavenging endogenous molecules produced by dying cells in normal or pathological conditions, usually leading a controlled and self-limited sterile inflammatory response [7].

It has been also unveiled TLRs role in repairing damaged tissue beyond antimicrobial defense, as in the case of ischemia reperfusion injury [8]. In noninfectious inflammatory diseases such as atherosclerosis and Alzheimer’s disease, oxidized low-density lipoprotein and amyloid, respectively, trigger sterile inflammation [9]. The activation of TLR signaling during tissue damage suggests that endogenous molecules serve as TLR agonists for maintenance of homeostasis, such as tissue repair [7]. Therefore, TLRs may participate in ocular surface immune homeostasis and their changes within the age might also modify ocular surface para-inflammatory equilibrium causing its age-related dysregulation, hence inflammAging [10].

Then, our aim in the present study was to evaluate age-related TLR changes at the ocular surface of “healthy” volunteers, and its impact on tissue inflammAging.

## Methods

Fifty-one (51) consecutive volunteers were enrolled in an open observational pilot study at Ophthalmology Complex Operative Unit (University Campus Bio-Medico, Rome, Italy). Volunteers were categorized into 3 groups according to age: young (22–40 years), middle-aged (41–60 years) and elder (61–85 years) [2, 4]. Eligible subjects were at least 18 years of age and described themselves as “healthy”. Exclusion criteria included history and/or signs/symptoms of ocular diseases, contact lens use and previous ocular surgery. Systemic cardiovascular, metabolic, neoplastic and psychiatric diseases as well as pregnancy were further exclusion criteria. In addition, all patients using any topical and/or systemic medications within the three-month period preceding the examination were excluded.

All study procedures were performed in accordance with guidelines established by the Association for Research in Vision and Ophthalmology, reviewed and approved by the Intramural Ethical Committee (University Campus Bio-Medico, Rome, Italy), observing the tenets declaration of Helsinki. All participants provided a written informed consent to proceed for clinical and laboratory analysis.

### Clinical assessment

Ocular surface examination included slit lamp assessment of ocular surface, Tear Break-Up Time (T-BUT), Schirmer Test Type 1, and administration of Ocular Surface Disease Index (OSDI) questionnaire to collect symptoms related to ocular surface irritation and/or discomfort, according to a standard procedure [11].

OSDI is a 12-item validated questionnaire designed to provide a rapid assessment of symptoms related to ocular surface irritation and their impact on vision-related functioning in patients with dry eye disease (13–22 mild, 23–32 moderate and over 33 severe).

### Conjunctival imprints

Two impression cytologies (ICs) from each bulbar conjunctiva (temporal and nasal ones) were sampled using a Millicell filter (0.22 μm membrane; Millipore, Milan, Italy) [2]. One imprint was citofixed for immunofluorescence and the other was extracted for molecular analysis.

Plasticwares and analytical grade chemicals were purchased from EuroClone (Milan, Italy), Sigma Aldrich (St. Louis, MO, USA) and Carlo Erba (Milan, Italy). Other reagents are specified in the text.

### Relative real-time PCR

Total RNA was extracted from no-pooled ICs by using the Mirvana Paris extraction procedure (AM1556; mirVana™ PARIS™ RNA and Native Protein Purification Kit; Thermo Fischer Scientific, Massachusetts, USA) with minor modifications. RNA was resuspended in 15μL MilliQ water (autoclaved DEPC-treated RNase free water; Direct Q5, Millipore Corporation, Billerica, MA, USA). To remove any DNA contamination, rehydrated RNA samples were treated with RNase-free DNaseI (2U/μLDNAse) following the kit instructions (AM1907; Turbo DNA free kit, Ambion; Thermo Fisher Scientific). RNA (purity > 1.8 and quantity) analysis was provided by SPECTROstar Nano (BMG LABTECH GmbH, Allmendgrün, Ortenberg). Equivalent (500 ng) RNA amounts were used to synthesize cDNAs by GoScript™ Reverse Transcription kit (Promega, Madison, Wisconsin, USA) in a programmable thermocycler (LifePRO/BIOER; Euroclone, Milan, Italy). Specific PCR amplifications were carried out by using the SYBRgreen Hot-start PCR master Mix (Hydra; Biocell, Rome, Italy) in the presence of cDNA templates and specific primer pair (one intron-spanning; Primer3web version 4.1.0; synthesis by Eurofins Genomics (Biotech, Ebersberg, Germany)). Appropriate amplification parameters are summarized in Table 1. Amplifications were performed in 48-well microplates (real-time PCR Eco™ Illumina; San Diego, CA). Single cycle threshold values (Ct) were recorded at the end of each amplification and normalized to those of referring genes run in parallel (nCt = Ct_target_ − Ct_referring_).[12, 13] Fold changes (2log) between subgroups were calculated according to the ΔΔCt and 2^-ΔΔCt analysis (REST384–2006 software) [14]. Single band PCR products (100-300bps) were randomly verified in 2.5% agarose—FluoVis gel (SMOBIO technology Inc., Hsinchu City, Taiwan).

**Table 1:** Primer description

*Genes*	*Primer pairs (F/R)*	*Annealing temp. °C*
*Referring genes*		
*H3*	F: 5'-ACT CAT CAC CGT AGC CAG GT-3' R: 5'-CAC CTT GGC TTG AGC ACT C-3'	60 °C
*18S*	F: 5’-GGA GAG GGA GCC TGA GAA C-3’ R: 5’-AGG GCC TCG AAA GAG TCC T-3’	58 °C
*Target genes*		
*TLR-1*	F: 5'-TTC ACA GTG TCT GGT ACA CGC AT-3' R: 5'-ACC GTG TCT GTT AAG AGA TTA TTG GA-3'	58 °C
*TLR-2*	F: 5'-AGT GAG CGG GAT GCC TAC T-3' R: 5'-GAC TTT ATC GCA GT CTC AGA TTT AC-3'	60 °C
*TLR-3*	F: 5'- GCC GCC AAC TTC ACA AGG TAT A-3' R: 5'- AGC TCA TTG TGC TGG AGG TT -3'	58 °C
*TLR-4*	F: 5'-AAT CCC CTG AGG CAT TTA GG-3' R: 5'-CAG GGC TAA ACT CTG GAT GG -3'	58 °C
*TLR-5*	F: 5'-CCA TAG ATT TTT CCT CCA ACC AAA TA-3' R: 5'-TCA TAC ATT TTC CCC AGT CCA CT-3'	58 °C
*TLR-6*	F: 5'-CAT CCT ATT GTG AGT TTC AGG CAT-3' R: 5'-GCT TCA TAG CAC TCA AC CCA AG-3'	59 °C
*TLR-7*	F: 5'-GGA GGT ATT CCC ACG AAC ACC-3' R: 5'-TGA CCC CAG TGG AAT AGG TAC AC-3'	60 °C
*TLR-8*	F: 5'- AAA CTT GAC CCA ACT TCG ATA CCT AA-3' R: 5'-GAT CCA GCA CCT TCA GAT GAG G-3'	60 °C
*TLR-9*	F: 5'-TTC ATG GAC GGC AAC TGT TA-3' R: 5'-GAG TGA CAG GTG GGT GAG GT-3'	58 °C
*TLR-10*	F: 5'-AAG AAA GGT TCC CGC AGA CTT-3' R: 5'-TGT TAT GGC ATA GAA TCA AAA CTC TCA-3'	58 °C

*TLR* Toll-like Receptor, *H3* Histon 3, *18S* 18S ribosomal RNA

Details in primer design/synthesis and amplification set are shown in the text (M&M)

### Immunofluorescence and epifluorescent microscopy

Cytofixed ICs (Citofix, Bio-Optica, Milan, Italy) were quickly pretreated for 20 min with 0.8% bovine serum albumin – 0.3% Triton X100 in 0.01 M Phosphate-Buffered Saline (PBS) before the addition of primary antibodies diluted at 1 µg/ml in PBS. The primary antibodies were as follows: the polyclonal anti-TLR2 (sc-8689; 1 µg/ml; raised in goat), anti-TLR3 (sc-10740; raised in rabbit), anti-TLR4 (sc-10741; raised in rabbit), anti-TLR5 antibodies (sc-8695; raised in goat), anti-TLR7 (sc-13208; raised in goat) and anti-TLR9 (sc-16247; raised in goat) antibodies and the monoclonal anti-ICAM1 antibodies (sc-18853; raised in mouse), used as and purchased from Santa Cruz Biotech (Santa Cruz, CA). For labeling, cy2 (green) or cy3 (red)—conjugated specie-specific secondary antibodies were used (1:300; Jackson ImmunoResearch, West Grove, PA) followed by nuclear staining with DAPI (5 µg/mL 4’,6-Diamidino-2-phenylindole; Molecular Probes, Invitrogen, Milan, Italy) diluted in PBS supplemented with 20 µg/mL RNAse (Molecular Probes). Appropriate isotype-matched IgG staining (Vector Labs. Ltd., Burlingame, CA) were performed in parallel. The direct epifluorescent microscope (Eclipse Ni) equipped with plan fluor X20/0.50 and X40/0.75 objectives, and plan Apo X60/1.40 objectives (Nikon) was used for the observations and digital images were produced by using a digital camera (Axiocam 208 color) and the free available ZEN 3.1 blue edition software (Carl Zeiss, Jena, Germany). For quantification purpose, single-channel images were analysed upon background subtraction. Defined optic field areas were selected to ensure areas equally cell populated (Image J v1.43; NIH -http://rsb.info.nih.gov/ij/). Mean Fluorescence Intensity (MFI; mean ± SD) values were used for graphical representation (GraphPad Software; Prism 8, San Diego, CA). Representative images were assembled in panels by using the Adobe Photoshop software (release 2020 22.0.0; Adobe Systems Inc., San Jose, CA).

### Statistical analysis

Data values are expressed as mean ± SD (text) and mean ± SEM (graphics). The statistical package used was StatView II for PC (Abacus Concepts. Inc., Barkley, CA, USA). Differences between groups were assessed with ANOVA followed by a Tukey–Kramer post hoc analysis. Correlations between two variables were evaluated by Pearson’s correlation coefficient analysis. P ≤ 0.05 was selected as the limit of statistical significance.

## Results

**Fig. 1:**
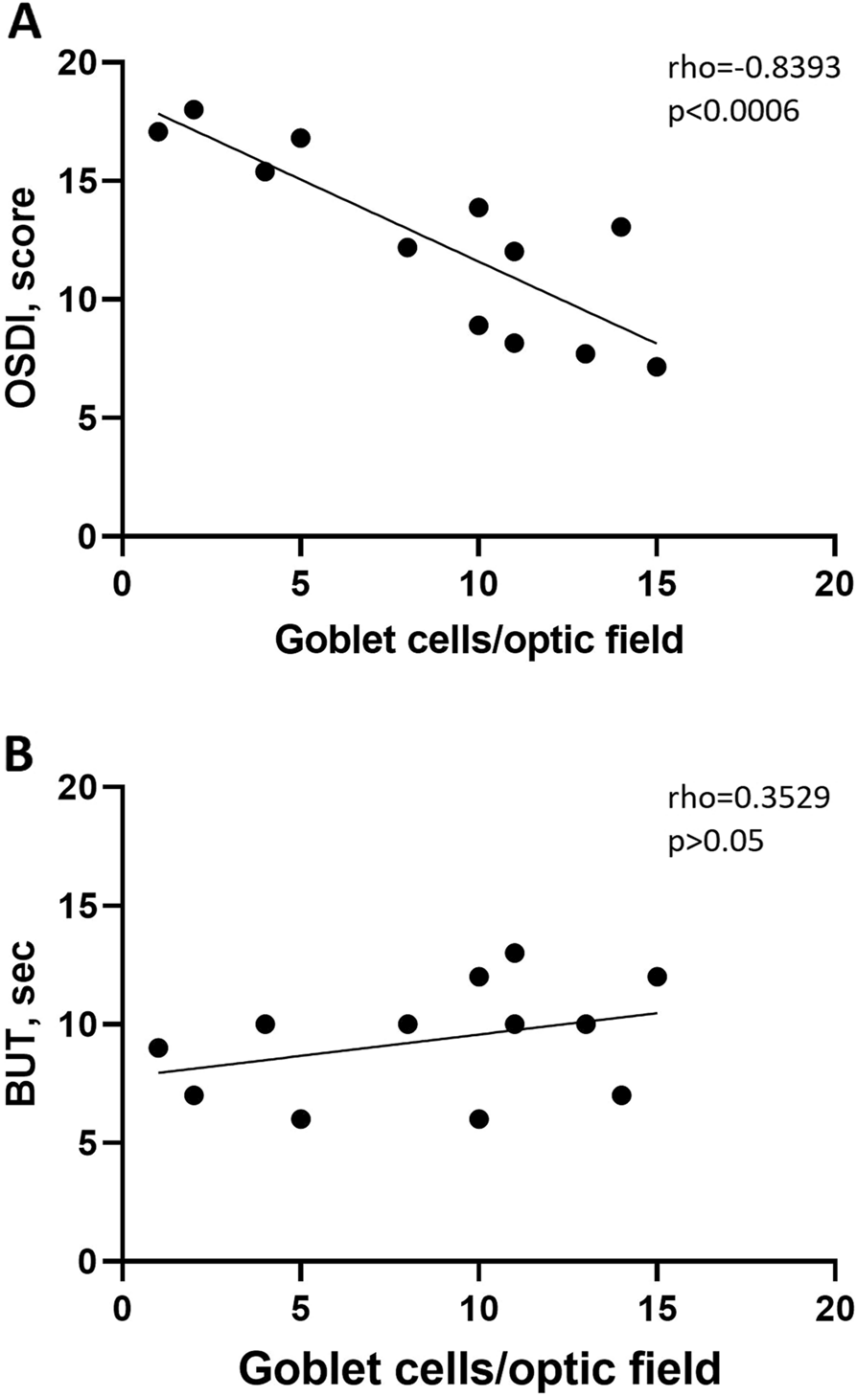
Clinical assessments of ocular surface system function. Goblet cells number correlate negatively with OSDI (**A**) and positively with T-BUT (**B**). Pearson post hoc: *R* and *p* values are reported in the panels

Clinical assessments revealed OSDI scores (young: 7.65 ± 0.71score; middle age: 12.54 ± 0.71 score; old: 17.40 ± 0.85 score; *p* < 0.05), hence ocular discomfort perception, significantly increased with ageing, and correlated negatively with Goblet cells number (*R* = -0.8393, *p* < 0.0006 Fig. 1 A). By contrary, BUT values, which is direct marker of tear film stability, decreased with ageing (young: 11.50 ± 1.81 s; middle age: 8.75 ± 1.50 s; old: 8.17 ± 2.50 s; *p* < 0.001) and positively correlated with Goblet cells number (rho = 0.3529, *p* > 0.05 Fig. 1 B) as well as type I Schirmer test (young: 20.07 ± 0.74 mm; middle age: 17.85 ± 1.34 mm; old: 14.65 ± 0.92 mm, *p* < 0.05), unveiling an ocular surface system and tear film failure in such elder population. Demographics are reported in Table 2.

**Table 2:** Study population

*Groups*	*N°*	*Age*	*Age* ± *SD*	*Sex*
*Population*	51	22–85	49.57 ± 3.76	27F/24 M
*Young*	18	22–40	25.32 ± 1.17	10F/8 M
*Middle-age*	16	41–60	49.87 ± 5.31	6F/10 M
*Old*	17	61–85	73.47 ± 8.68	11F/6 M

**Fig. 2:**
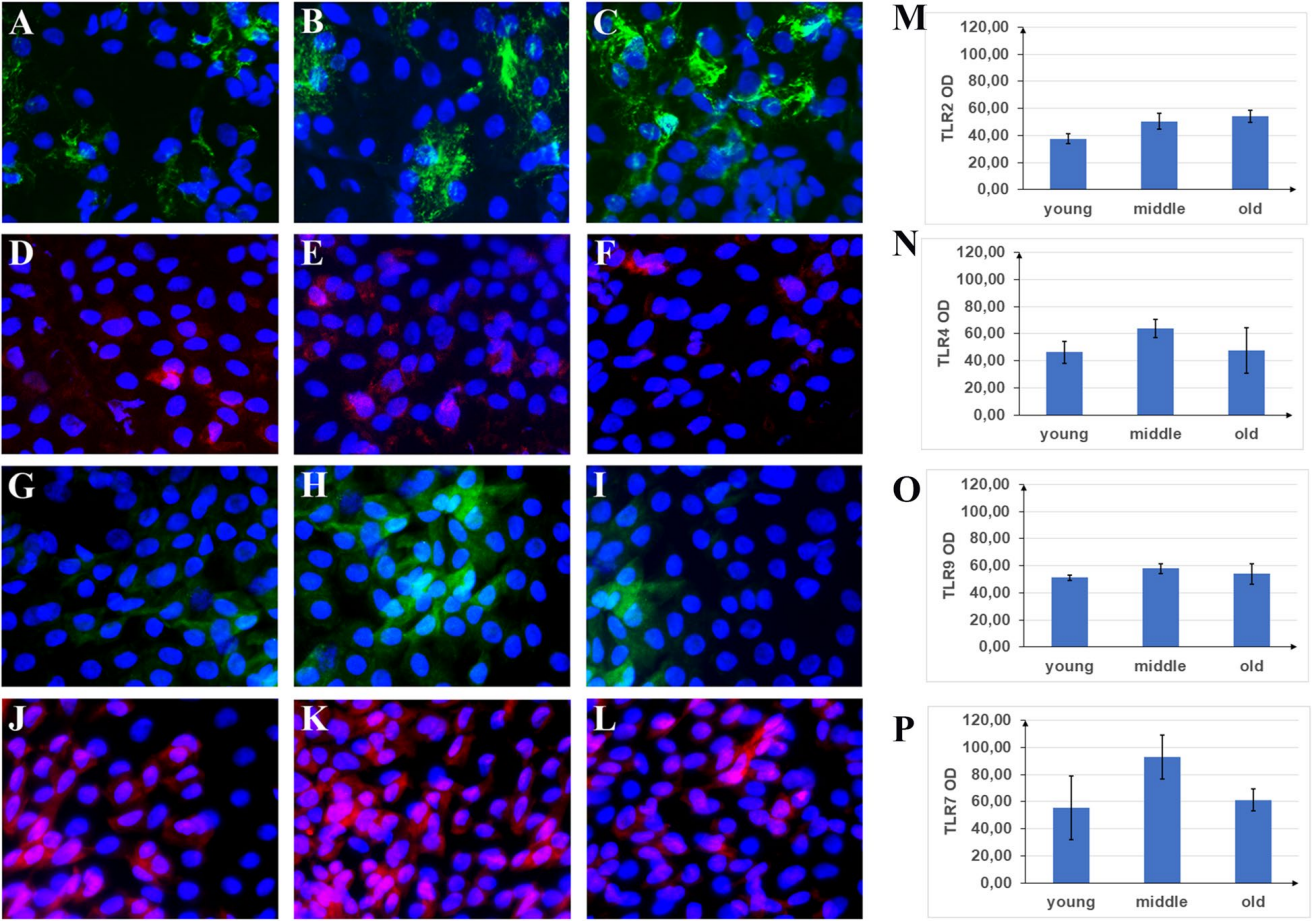
TLR immunoreactivity in conjunctival imprints. Imprints were cytofixed and directly immunostained to assess protein expression by epithelial monolayers. Epifluorescence acquisitions (overlays) of TLR immunolabeled (green/cy2 or red/cy3) imprints over a blue nuclear counterstaining (dapi/blue) are shown. Representative merge panels showing the specific TLR2 (**A**-**C**), TLR4 (**D**-**F**), TLR9 (**G**-**I**) and TLR7 (**J**-**L**) immunoreactivities respectively in young (left), middle age (middle) and old (right) panels. IntDen values are reported in bar plots respectively for all TLR immunostained (M-P): an increase of TLR2 was observed by ageing; a significant increase of TLR4 immunoreactivity was observed in middle age. Epifluorescent layers were acquired on the same optic field and no color adjustmente were performed for figure preparation. Scale bar = 10 µm (bar in the panel). Immunofluorescence analysis (*n* = 4) at × 40/dry 0.75 DIC M/N2 magnification, 300 dpi resolution (1920 × 1080 pixels)

TLR-2 immunoreactivity increased significantly with ageing (young: 37.62 ± 3.58 IntDen; middle age: 50.50 ± 5.67 IntDen; old: 54.22 ± 4.27 IntDen) (Fig. 2 A-C, M).

TLR4 immunoreactivity showed a significant increase at middle age: (young: 46.39 ± 8.07 IntDen; middle age: 63.69 ± 6.66 IntDen; old: 47.77 ± 16.69 IntDen) (Fig. 2 D-F, N) as well as TLR-9 (young: 51.30 ± 2.00 IntDen; middle age: 57.94 ± 3.54 IntDen; old: 54.01 ± 7.37 IntDen) (Fig. 2 G-I, O) and TLR7 (young: 55.68 ± 23.53 IntDen; middle age: 92.94 ± 16.35 IntDen; old: 61.24 ± 7.98 IntDen) (Fig. 2 J-L, P); TLR5 values were similar in all groups (data not shown).

**Fig. 3:**
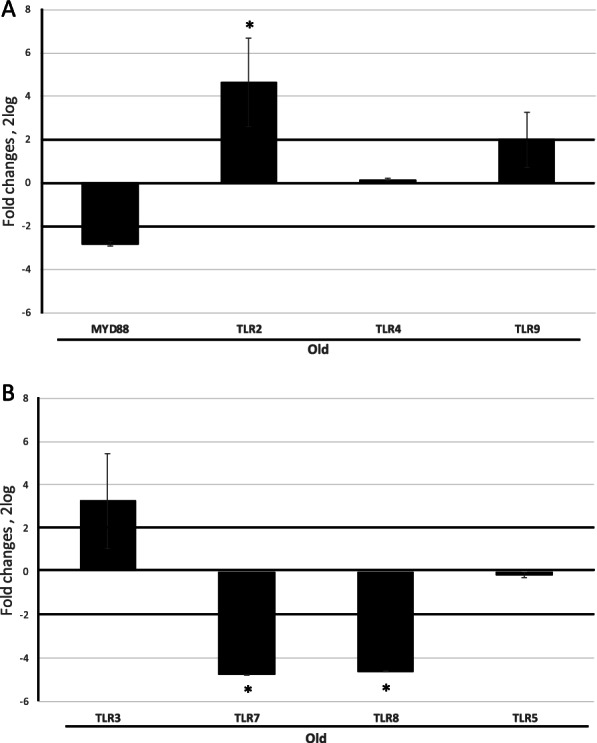
Changes in TLRs transcript expression at ageing. Total RNA was extracted by conjunctival imprints and cDNA was synthetized for specific amplification in relative real time PCR platform. All comparisons were performed against young imprints (REST analysis followed by Tukey–Kramer post hoc). **A** A significant upregulation of TLR2 was observed in old imprints, while no significant changes were detected for TLR4 and TLR9. **B** In old imprints, a significant deregulation was observed for TLR7 and TLR8, while TLR5 was unchanged. Asterisks (*) in histograms point at significant differences between subgroups (*p* < 0.05). Dashed line indicate range significance for REST analysis (2log comparisons)

A decreasing reactivity was observed for TLR3 with age (young: 73.61 ± 1.28 IntDen, middle age 62.34 ± 5.78 IntDen, old: 56.25 ± 5.45 IntDen; Fig. 3 B).

**Fig. 4:**
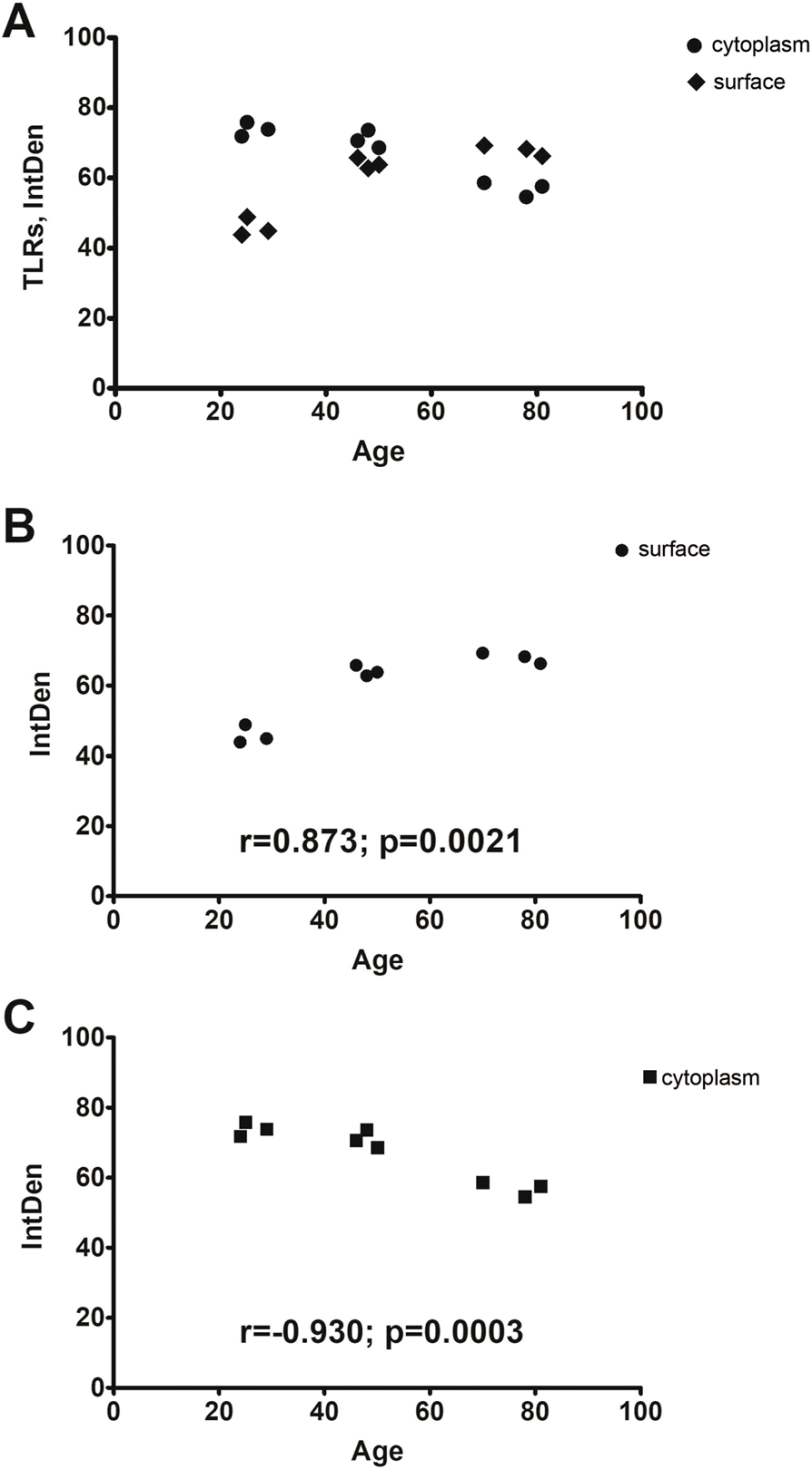
Age-related TLRs changes: a comparison between cell surface and intracellular expressions. Epifluorescent TLR immune-labeled imprints were subjected to IntDen analysis, according to the ImageJ software, after channel split and background normalization (single channel; × 200). Overall IntDen value distribution is shown in panel (**A**). Correlation analysis showed a significant increasing expression of surface TLRs (TLR2 and TLR4) (**B**) with ageing associated to a reduction of cytoplasmic TLRs (TLR7 and TLR9) (**C**). Pearson post hoc: *R* and *p* values are reported in the panels

**Fig. 5:**
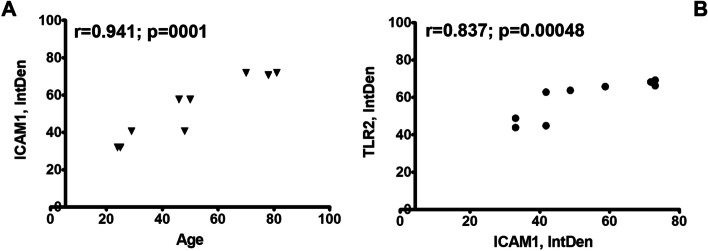
TLR2 and InflammAging. ICAM-1 immunoreactivity in conjunctival imprints was quantified by IntDen analysis. ICAM-1 expression increased with ageing (**A**), supporting the biomolecular inflammAging changes in our population. Increasing ICAM-1 correlated positively with increased TLR2 expression, highlighting TLR2 roles in aged ocular surface (**B**)

A molecular evaluation of imprints was performed and the REST analysis of middle-age and old transcripts, with respect to young one, is shown in Fig. 3 A-B. The analysis showed a significant downregulation of TLR4 (-2.55 ± 0.09 2log-fold changes; *p* < 0.05; Fig. 3 A) and a trend to a decrease of MyD88 (-2.83 ± 0.05 2log-fold changes; Fig. 3 A). Upregulation of TLR2 (4.65 ± 2.05 2log-fold changes, *p* < 0.05) in old population was also evaluated as compared to young one (Fig. 2 A). TLR7 (-4.76 ± 0.01 2log-fold changes, *p* < 0.05) and TLR8 (-4.61 ± 0.01 2log-fold changes, *p* < 0.05) transcripts were significantly downregulated in old population (Fig. 3 B). Not significant changes were observed for TLR9 and TLR5 transcripts (Fig. 3 A-B). An overall increase of cell surface TLRs and a decrease of intracellular ones with ageing was also detected significantly (Fig. 4). A direct correlation was showed for increasing ICAM-1 (Fig. 5 A) and ageing as well as increasing TLR2 immunoreactivity (Fig. 5 B).

## Discussion

Toll like receptors at ocular surface of healthy people changed with the age. Increased expression of cell-surface TLRs with downregulation of cytoplasmatic TLRs was detected. Increasing TLR2 and reducing TLR3 and 7 expression was strictly correlated with increasing age-related expression of ICAM-1, then inflammation.

Clinical (T-BUT, Schirmer Test Type 1, and OSDI) and biomolecular (ICAM-1 and goblet Cells) ocular surface function markers unveiled an unperceived inflammAging in our older volunteers [2]. Such clinical and biomolecular changes were significantly correlated to increased TLR2, and reduced TLR7-8 in elder population, unveiling a role of TLRs in ocular surface homeostasis, and not only in microbial defense [15].

Such “ageing patterns” may cause alteration of local immunity [2]. Age-related alteration of TLRs local expression may be a preclinical and/or subclinical condition that participates to the failure of the para-inflammatory compensatory mechanisms which keep ocular surface homeostasis [7, 10, 15–19].

TLRs are primary components of the innate immune system involved in signal transduction and are expressed by entire “healthy” ocular surface, allowing a prompt innate response to pathogenic strains that might trigger local inflammation [19]. The human ocular surface is colonized by an expansive varied microbial community, as evidenced by previous researches, in equilibrium state [10, 15–23]. TLRs are taking part in controlling such homeostatic balance at the ocular surface, bridging the gap between microbial flora and ocular surface.

However they are part of family of membrane-bound pattern recognition receptors (PRRs) located either on the cell surface or in endosomal compartments. These receptors are known to respond to host-molecules termed damage-associated molecular patterns (DAMPs), which act as ‘‘non-self’’ antigens. [24]

The eye normally exhibits ‘‘immune and angiogenic privilege’’ to avoid the potential sight-threatening consequences of ocular inflammation to antigens [25]. Such privilege involves anatomical, cellular, and soluble factors, as well as TLRs, ensuring homeostatic equilibrium [26]. however this state of privilege may break down, resulting in a dysregulated sterile inflammatory status, with age [27].

Sterile inflammation does not show signs of acute inflammation; rather it appears to be low-grade and chronic. Sterile inflammation occurs in response to a growing list of modified host-derived elements ranging from oxidized lipids or lipoproteins to deposits of protein/lipid aggregates or particulate matter [24]. These stimuli trigger activation of PRRs of the innate immune system, such as TLRs [28].

In fact frequently these stimuli are often easily cleared, leading to a self-limiting and controlled homeostatic para-inflammation. But, once the system is dysregulated, the inflammatory response can persist causing over-activation of the immune system and contributing to disease pathogenesis [2, 29, 30].

In fact, as per our previous data[2], with progressive age, increased oxidative damage occurs in many other tissues, including the retina, contributing to the multiple forms of retinal degeneration most notably age-related macular degeneration (AMD) [29].

TLR2 has an alert sentinel function and is the main recruitment/phagocytic receptor for innate cells in case of bacterial infection[31], and it is also involved in promoting angiogenesis during wound healing [32, 33].

Excessive ROS can damage lipids through a mechanism known[29] and conceivably induce activation of TLR2 in AMD and in other retinal diseases where ROS play a role in pathology TLR2 inhibition provides striking protection to the retina in response to oxidative stress [29]. TLR2 mediates complement deposition in response to oxidative stress, then blocking TLR2 signaling preserves cells integrity in vivo under conditions of acute oxidative stress [29, 33, 34].

Therefore at ocular surface with ageing the absolute and relative increase of TLR2 is directly related to increasing ICAM expression and, hence, inflammation, suggesting a TLRs disequilibrium. TLRs unbalance may be contributing cause of the altered homeostatic mechanisms occurred with ageing.

Healthy corneal epithelium usually do not express TLR2 at the cell surface, failing to elicit immune response to ligands (TLR silent form) [35], but expresses high levels of TLR9, compared with the average expression of TLR-2, whereas the expression by the underside stroma is at similar levels [36]. Therefore the overexpression of cytoplasmatic TLRs may create an immunosilent condition to prevent unnecessary inflammatory responses [35]. A relative reduction of cytoplasmatic TLR7/8 and TLR9, in old subjects may trigger the activation of the innate as well as adaptive immune response [37–39].

In fact, although still controversial and debated, several evidences describe the immunomodulating activity of cytoplasmatic TLRS, such as TLR7, 8, 9 and 3, underlining their role in inducing immunotolerance [40–44].

Therefore, an increased expression of TLR2 and a relative decrease of cytoplasmatic TLRs compared to younger population may expose elder population to inflammAging.

Finally ageing has been associated with several ocular dysfunctions and degenerations such as (1) ocular surface failure which may worsen in in dry eye disease and post-surgery discomfort syndrome, (2) Age related macular degeneration. Both conditions are related to a loss of homeostatic mechanisms, and dysregulated parainflammation leading to a tissue failure and sterile subclinical persistent inflammation. This inflammAging at ocular surface may be partially caused by a TLRs expression and signaling unbalance.

## Conclusions

Age related variations in TLRs expression in apparently healthy volunteers may influence local immune surveillance and para-inflammatory homeostatic response. These new insights unveil the complexity of the inflammAging but also the critical role of TLRs in in regulating the immune response, hence autoimmunity, at ocular surface.

## Data Availability

The data used to support the findings of this study are available from the corresponding author upon request.

## Abbreviatons

TLR: toll-like receptor
ER: endoplasmic reticulum
APC: antigen presenting cells
T-BUT: tear break-up time
OSDI: ocular surface disease index
IC: impression cytology
PCR: Polymerase Chain Reaction
Ct: cycle threshold
PBS: phosphate buffered saline
IntDen: integrated densitometric
FI: fluorescence intensity
